# Multimodality imaging characterization of Perugini scintigraphic grades in transthyretin amyloid cardiomyopathy: a single-center experience

**DOI:** 10.1186/s44348-026-00075-8

**Published:** 2026-06-17

**Authors:** Paulius Bucius, Evelina Zarambaite, Kornelija Lusaite, Matas Streckis, Arnas Karuzas, Jurgita Plisiene, Gintare Sakalyte, Tomas Lapinskas, Donatas Vajauskas, Antanas Jankauskas, Egle Ereminiene

**Affiliations:** 1 Institute of Cardiology, Lithuania University of Health Sciences, Kaunas, Lithuania; 2 Heart Center, Medical Academy, Lithuanian University of Health Sciences, Kaunas, Lithuania; 3 Department of Radiology, Medical Academy, Lithuanian University of Health Sciences, Kaunas, Lithuania

**Keywords:** Amyloidosis, Cardiomyopathies, Echocardiography, Magnetic resonance imaging, Biomarkers

## Abstract

**Background:**

Transthyretin amyloid cardiomyopathy (ATTR-CM) has undergone a shift towards noninvasive diagnostics using bone tracer scintigraphy with Perugini grading. While both grades 2 and 3 are considered diagnostic, potential phenotypic differences between these groups remain uncertain. We aimed to evaluate the diagnostic yield of technetium-99 m pyrophosphate (99mTc-PYP) scintigraphy and to compare clinical and multimodality imaging characteristics across scintigraphic grades in a single-center cohort.

**Methods:**

We retrospectively reviewed all patients who underwent 99mTc-PYP scintigraphy for suspected cardiac amyloidosis between 2018 and 2025. Patients with confirmed ATTR-CM were stratified by Perugini grade (grade 2 vs. grade 3). Clinical features, biomarkers, electrocardiography, echocardiography, and cardiac magnetic resonance (CMR) parameters were compared. Correlations between scintigraphic grade, imaging markers, and biomarkers were assessed.

**Results:**

Among 302 scans, 103 (34.1%) had reported cardiac uptake on planar imaging. Fifty-three patients were diagnosed with ATTR-CM, including 8 (15.1%) with grade 2 and 45 (84.9%) with grade 3 uptake. Grade 3 patients exhibited worse functional status, shorter 6-min walk distance, and higher troponin I levels. Echocardiography showed greater maximal wall thickness, lower left ventricular ejection fraction, and worse global longitudinal strain. In the subset with CMR (*n* = 38), grade 3 patients (*n* = 30) had significantly higher left ventricular mass index, extracellular volume fraction, right ventricular free wall thickness, and more frequent right ventricular involvement. Perugini grade showed strong rank-based correlations with left ventricular mass index (ρ = 0.61, *P* < 0.01) and extracellular volume (ρ = 0.53, *p* < 0.01), and a moderate correlation with troponin I (ρ = 0.55, P < 0.01). A modest correlation with brain natriuretic peptide (BNP) was also observed (ρ = 0.36, *P* < 0.05), although BNP levels did not differ significantly between grade groups. Grade 1 uptake was not associated with subsequent ATTR-CM diagnosis on available follow-up.

**Conclusions:**

Noninvasive scintigraphy provides effective detection of ATTR-CM in a referral population. Although grades 2 and 3 both meet diagnostic criteria, in this retrospective cohort, grade 3 uptake was associated with markers of more advanced structural remodeling, higher extracellular volume, greater right ventricular involvement, and worse functional impairment.

## Background

Transthyretin amyloid cardiomyopathy (ATTR-CM) has become an increasingly recognized cause of heart failure with preserved ejection fraction. Historically, its diagnosis relied on endomyocardial biopsy, the risks and limited availability of which often outweighed the potential clinical benefit, especially given the historical lack of specific treatments. However, with the validation of a noninvasive, nuclear medicine-based diagnostic approach [1, 2] and the increasing availability of disease-modifying agents [3–5], the paradigm has shifted towards the identification of early signs of the disease [6].

The interpretation of scintigraphic studies is based on the Perugini grading system, a visual scoring method comparing myocardial uptake to the surrounding ribcage [7]. Current guidelines classify grade 2 and grade 3 uptake as positive for ATTR-CM, provided that the presence of monoclonal gammopathy has been excluded. While both grade 2 and grade 3 are diagnostic, there is ongoing debate regarding phenotypic and prognostic differences between these two groups [8, 9].

In this study, we present a 7-year single-center experience in diagnosing ATTR-CM. We aim to describe the volume of disease detection through technetium-99 m pyrophosphate (99mTc-PYP) scintigraphy, as well as the clinical and imaging characteristics of our patient population. Furthermore, we sought to explore whether Perugini grade 2 and grade 3 uptake were associated with different clinical, biomarker, echocardiographic, and cardiac magnetic resonance (CMR) phenotypes in a real-world referral cohort.

## Methods

### Ethics statement

The study was approved by the Kaunas Regional Bioethics Committee (No. BE-2–50) and was performed in accordance with the Declaration of Helsinki. Informed consent was obtained from all individual participants included in the ATTR-CM cohort, while informed consent was waived for participants whose data was collected retrospectively.

### Study design

In this study, we reviewed all 99mTc-PYP scintigraphy scans performed for suspected cardiac amyloidosis at Lithuanian University of Health Sciences Hospital Kauno Klinikos. Clinical and imaging data temporally closest to the scintigraphy scan were used for further analysis.

### 99mTc-PYP scintigraphy

**Fig. 1:**
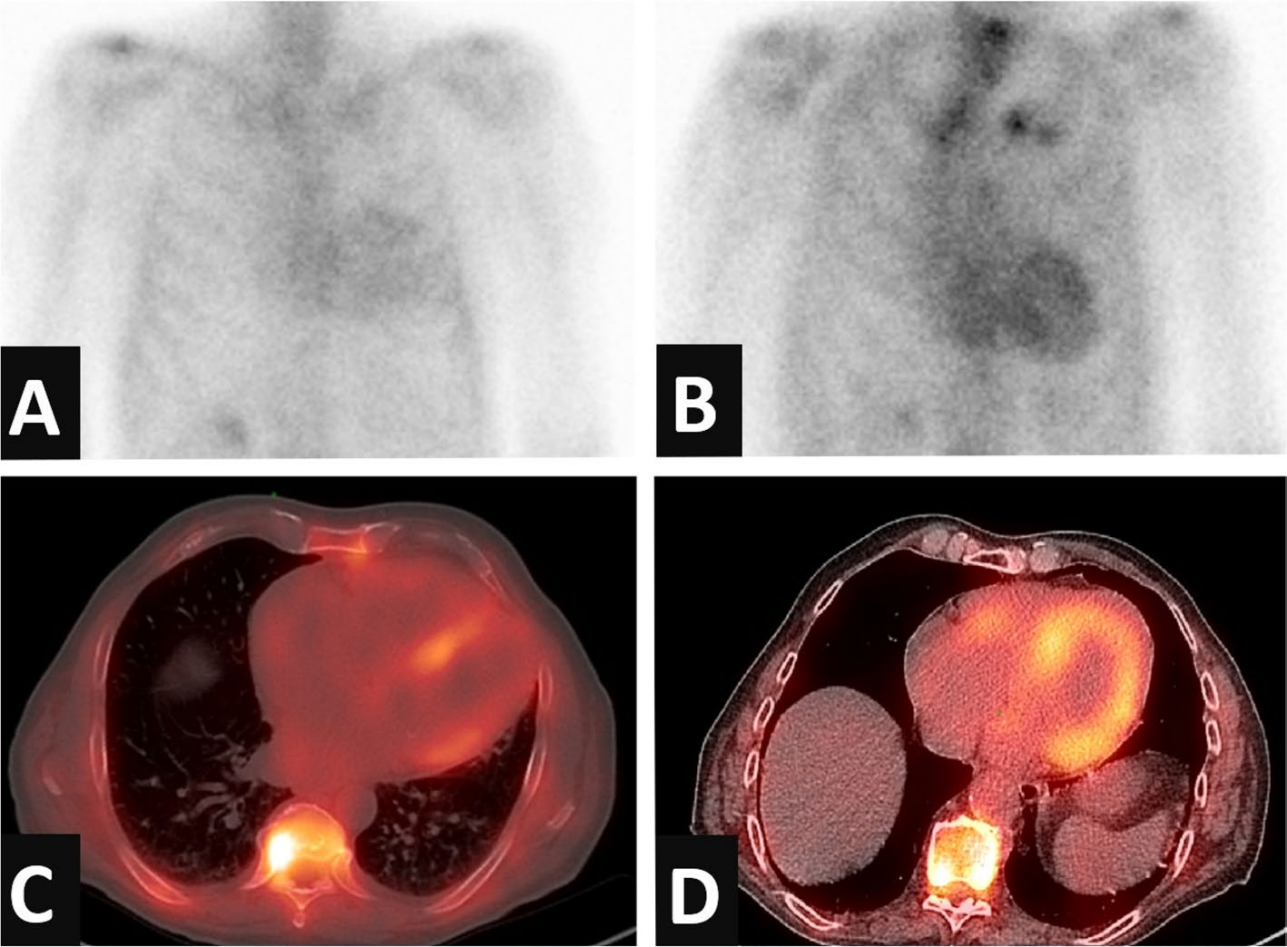
Scintigraphy with technetium-99 m pyrophosphate. **A**, **B** Planar scintigraphy images. **C**, **D** Single-photon emission computed tomography/computed tomography fusion images. **A**, **C** Images of a patient with Perugini grade 2 uptake. **B**, **D** Images of a patient with Perugini grade 3 uptake

**Fig. 2:**
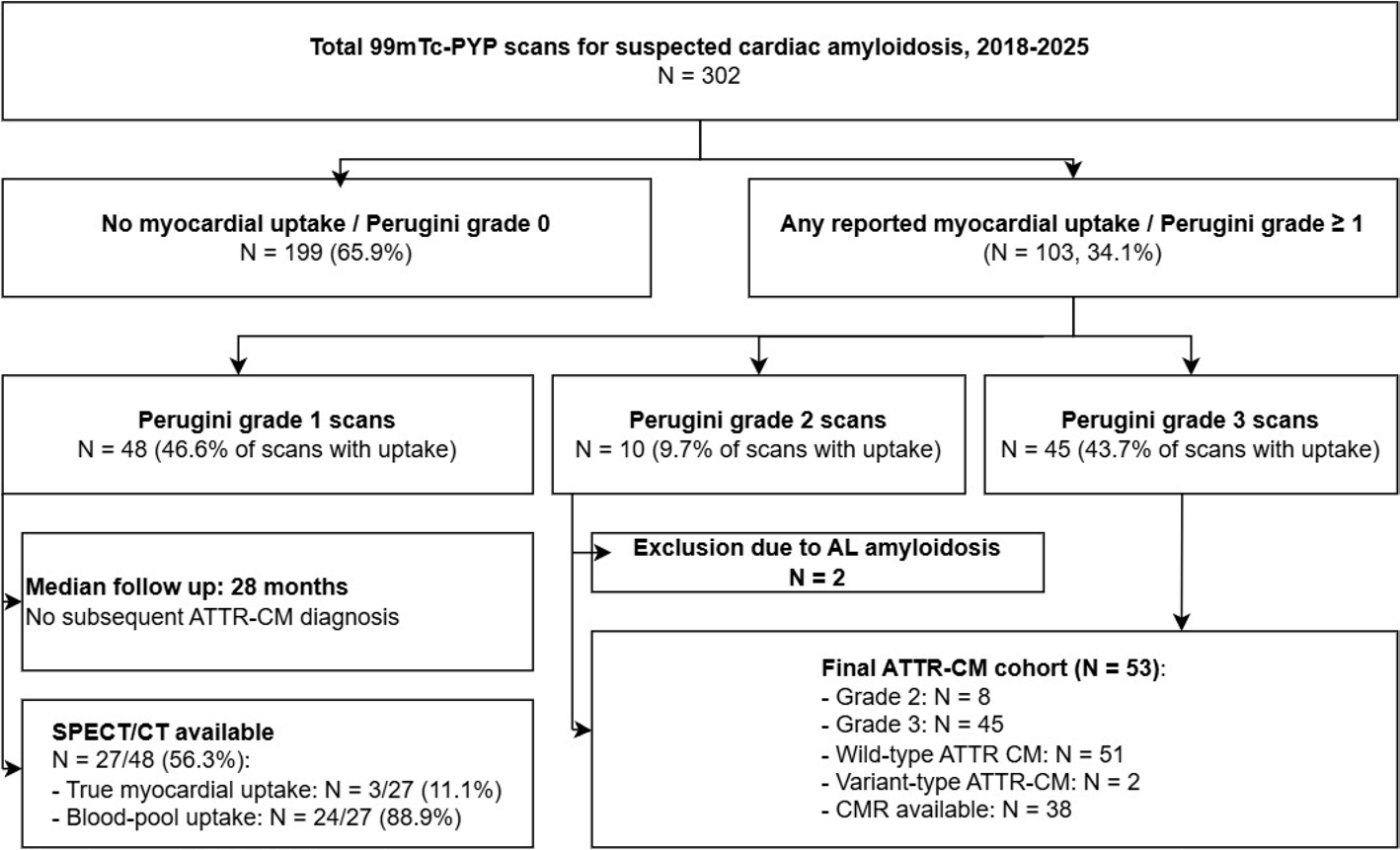
Study flowchart showing patient selection, scintigraphic classification, and final transthyretin amyloid cardiomyopathy (ATTR-CM) cohort. Cardiac magnetic resonance (CMR) was available in 38 patients. 99mTc-PYP, technetium-99 m pyrophosphate; AL, amyloid light chain; CT, computed tomography; SPECT, Single-photon emission computed tomography

All scintigraphy scans were performed using 99mTc-PYP on a hybrid single-photon emission computed tomography/computed tomography (SPECT/CT) scanner with 16-slice diagnostic CT (AnyScan, Mediso Medical Imaging Systems). Anterior and posterior planar views were obtained at 1 and 3 h after tracer injection. Where available, SPECT (early scan) and SPECT/CT (delayed scan) were used for confirmation of myocardial uptake (Figs. 1 and 2). All scans with clinically reported cardiac uptake (Perugini grade ≥ 1) were independently reviewed and regraded for the purposes of this study by one experienced nuclear medicine radiologist. Interobserver and intraobserver reproducibility analyses were not performed. All patients underwent systematic evaluation for monoclonal gammopathy using serum and urine immunofixation and serum free light chain assays [1].

### Electrocardiogram analysis

Electrocardiogram (ECG) recordings for all subjects diagnosed with ATTR-CM were reviewed by a cardiologist for the presence of conduction abnormalities, QRS width, low voltage, or pseudoinfarction pattern. Low voltage ECG was defined as either QRS amplitude < 10 mm in all precordial or < 5 mm in all limb leads. Pseudoinfarction pattern was defined as QS waves in leads V1–V3. Automatic analysis was used for determination of QRS width.

### Echocardiography

Echocardiographic studies were available in all patients and were performed within 3 months of the 99mTc-PYP scintigraphy scan. Two-dimensional echocardiography was performed by two experienced echocardiographers using an EPIQ 7 or Affiniti 70 ultrasound system (Philips Healthcare), in accordance with recommendations from the European Association of Cardiovascular Imaging [10]. Archived images were analyzed offline using Tomtec Imaging Systems software (Tomtec Imaging Systems).

Maximal wall thickness (MWT) was measured from parasternal long-axis view in B-mode images at end-diastole. Left ventricular ejection fraction (LVEF) was measured using the Simpson method from apical two- and four-chamber views. Left atrial–focused two- and four-chamber views were used for measuring left atrial volume index. LV global longitudinal strain (GLS) was determined from apical two-, three-, and four-chamber views using in-built applications. GLS values are reported as absolute values throughout the manuscript. Other parameters were measured directly from the acquired images.

### CMR analysis

All CMR examinations were performed within 4 months of the 99mTc-PYP scintigraphy scan. CMR scans were performed on a 3.0-T magnetic resonance imaging scanner (Magnetom Skyra, Siemens Healthcare) with an 18-channel cardiac coil. Cine images were acquired using a balanced steady-state free precession sequence in long-axis views (two-, three-, four-chamber views) and a short-axis stack during expiratory breath-holds and retrospective ECG gating. For T1 mapping, an ECG-gated single-shot modified Look-Locker inversion recovery (MOLLI) sequence with a 5(3)3 acquisition scheme was used. T1 mapping images were acquired in three short-axis slices (basal, mid-ventricular and apical) before contrast and 15 min after contrast injection (0.1 mmol/L gadobutrol [Gadovist]). Late gadolinium enhancement (LGE) sequences were acquired 10 min after contrast injection.

CMR images were analyzed with CMR post-processing software MEDIS Suite (Medis Medical Imaging) by a blinded observer. Standard volumetric techniques were used to determine LV and right ventricular (RV) volumes, as well as LVEF, RV ejection fraction (RVEF) and LV myocardial mass (LVM) parameters [11]. All volumetric parameters were indexed to body surface area. Short-axis CINE images were used for measuring maximal RV free wall thickness (RVFWT). Native T1 values were determined by delineating mid-myocardial layer of each myocardial segment. Hematocrit level for each patient was determined from a venous blood sample that was drawn immediately before the scan. A region of interest was drawn in the blood pool of before and after contrast T1 mapping images to create an extracellular volume (ECV) map. ECV values were calculated by measuring ECV in mid-myocardial layer of basal and mid-ventricular septum and then averaging the two results.

### Statistical analysis

Results are presented as either means ± standard deviations or absolute numbers with percentages. Study population was divided into two groups according to Perugini grade of PYP scan. Student t-test was used for normally distributed and Mann–Whitney U-test for non-normally distributed parameters. Fisher exact test was used to compare categorical data, given small group sizes and low expected cell counts. Correlations involving Perugini grade were assessed using Spearman rank correlation coefficient because Perugini grade is an ordinal variable. Correlations between continuous variables were assessed using Pearson or Spearman correlation coefficients according to data distribution. Analyses were performed using IBM SPSS ver. 22 (IBM Corp).

## Results

A total of 302 PYP scans were performed at our institution between 2018 and 2025. Of these, 103 (34.1%) had reported cardiac uptake on planar imaging. Uptake was distributed according to the Perugini grading system as follows: grade 1 (n = 48, 46.6% of scans with reported uptake), grade 2 (*n* = 10, 9.7%), and grade 3 (*n* = 45, 43.7%). Two patients with grade 2 uptake were confirmed to have amyloid light chain amyloidosis and were excluded from the analysis.

The remaining 53 patients with confirmed ATTR-CM comprised the final study cohort. This ATTR-CM population consisted of 8 patients (15.1%) with grade 2 uptake and 45 patients (84.9%) with grade 3 uptake. All patients included in the ATTR-CM cohort had clinical and/or echocardiographic findings that prompted evaluation for cardiac amyloidosis. Clinical features included heart failure symptoms, atrial fibrillation, conduction abnormalities, and extracardiac features commonly associated with ATTR-CM, including carpal tunnel syndrome and spinal stenosis. Echocardiographic findings included increased LV wall thickness, impaired GLS, apical sparing, and diastolic dysfunction, as summarized in Tables 1 and 2. Genetic analysis was performed in every patient and identified variant-type ATTR-CM in 2 patients (3.8%) and wild-type ATTR-CM in 51 patients (96.2%). The yearly distribution and diagnostic method for the cohort are presented in Table 3. ATTR-CM diagnoses increased over time, with 40 of 53 cases diagnosed during 2023–2025. Over the study period, the diagnostic pathway shifted from biopsy-based confirmation toward noninvasive diagnosis, which accounted for 45 of 53 diagnoses overall (84.9%) and more than 80% of annual diagnoses from 2023 onward.

**Table 1:** Comparison of clinical characteristics between grade 2 and grade 3 groups (n = 53)

Characteristic	Grade 2 (n = 8)	Grade 3 (n = 45)	P-value
Age (yr)	77.8 ± 4.9	78.2 ± 6.6	0.862
Sex			
Male	4 (50.0)	33 (73.3)	0.309
Female	4 (50.0)	12 (26.7)	
NYHA functional class			0.028^*^
I	1 (12.5)	2 (4.4)	
II	7 (87.5)	23 (51.1)	
III	0 (0)	19 (42.2)	
IV	0 (0)	1 (2.2)	
Pacemaker implantation	0 (0)	10 (22.2)	0.325^a^
Spinal stenosis	3 (37.5)	20 (44.4)	> 0.999^a^
Carpal tunnel syndrome	6 (75.0)	23 (51.1)	0.266^a^
Atrial fibrillation	4 (50.0)	33 (73.3)	0.246^a^
Paroxysmal	1 (12.5)	12 (26.7)	
Permanent	3 (37.5)	21 (46.7)	
Electrocardiographic feature			
Low voltage	2 (25.0)	14 (31.1)	> 0.999^a^
Pseudoinfarction	4 (50.0)	28 (62.2)	0.693^a^
Left bundle branch block	0 (0)	11 (24.4)	0.183^a^
Right bundle branch block	0 (0)	8 (17.8)	0.336^a^
QRS width (msec)	91.5 ± 7.9	119.8 ± 30.7	< 0.001^*^
BNP (ng/L)	139 ± 82	332 ± 341	0.120
Troponin I (μg/L)	0.025 ± 0.009	0.099 ± 0.090	< 0.001^*^
6MWT (m)	366 ± 38	298 ± 110	0.039^*^

Values are presented as mean ± standard deviation or number (%)

6MWT, 6-min walk test; BNP, brain natriuretic peptide; NYHA, New York Heart Association

^a^Calculated using the two-sided Fisher exact test

^*^P < 0.05

**Table 2:** Comparison of echocardiographic parameters between grade 2 and grade 3 groups (n = 53)

Parameter	Grade 2 (n = 8)	Grade 3 (n = 45)	P-value
LVEF (%)	53.8 ± 2.3	49.1 ± 8.2	0.003^*^
LVGLS (%)	16.0 ± 4.6	12.5 ± 3.9	0.037^*^
Apical sparing	4 (50.0)	40 (88.9)	0.021^a,*^
MWT (mm)	14.6 ± 2.3	16.9 ± 2.2	0.009^*^
LVEDDi (mm/m^2^)	24.7 ± 1.4	24.3 ± 2.6	0.460
LAVi (mL/m^2^)	48.2 ± 15.1	51.5 ± 16.1	0.620
E/e’	14.8 ± 3.4	17.6 ± 5.7	0.186
S’ (cm/sec)	12.8 ± 2.4	9.7 ± 3.1	0.010^*^
Pulmonary acceleration time (msec)	106 ± 12.6	94 ± 15.6	0.060
Pericardial effusion	0 (0)	10 (22.2)	0.325^a*^

Values are presented as mean ± standard deviation or number (%)

LAVi, left atrial volume index; LVEDDi, left ventricular end-diastolic diameter index; LVEF, left ventricular ejection fraction; LVGLS, left ventricular global longitudinal strain; MWT, maximal wall thickness

^a^Calculated using the two-sided Fisher exact test

^*^P < 0.05

**Table 3:** Annual number of ATTR-CM diagnoses and diagnostic modality during the study period (*n* = 53)

Year	No. of ATTR-CM diagnoses (%)		
	Total	Biopsy	Noninvasive
2018	1 (100)	1 (100)	0 (0)
2019	4 (100)	2 (50.0)	2 (50.0)
2020	2 (100)	0 (0)	2 (100)
2021	4 (100)	0 (0)	4 (100)
2022	2 (100)	1 (50.0)	1 (50.0)
2023	13 (100)	1 (7.7)	12 (92.3)
2024	12 (100)	2 (16.7)	10 (83.3)
2025	15 (100)	1 (6.7)	14 (93.3)
Total	53 (100)	8 (15.1)	45 (84.9)

ATTR-CM, transthyretin amyloid cardiomyopathy

To evaluate differences in disease presentation, the ATTR-CM cohort was stratified into groups of grade 2 (n = 8) and grade 3 (n = 45). The association between these scan grades and key clinical and imaging parameters was then assessed. To contextualize the diagnostic performance of scintigraphy, we additionally reviewed patients with Perugini grade 1 uptake. None of the patients with grade 1 uptake received a subsequent diagnosis of ATTR-CM during available follow-up (median, 28.0 months; interquartile range, 12.0–45.0 months). SPECT/CT imaging was available in 27 cases (56.2% of all grade 1 scans) and showed myocardial uptake in only 3 (11.1%) of these patients. The other 24 showed blood-pool uptake which was reported as myocardial uptake on planar imaging.

### Clinical and echocardiographic characteristics

Baseline clinical and echocardiographic characteristics are presented in Tables 1 and 2, respectively. The average age at diagnosis was 78 years (77.8 ± 4.9 years in grade 2 vs. 78.2 ± 6.6 years in grade 3, P = 0.862), and 69.8% of the cohort was male (50.0% vs. 73.3%, P = 0.309). Clinical features commonly associated with ATTR-CM, such as spinal stenosis (P > 0.999), carpal tunnel syndrome (P = 0.266), and atrial fibrillation (P = 0.246) were prevalent in the cohort, with no significant difference between the groups. While ECG features associated with cardiac amyloidosis (low voltage and pseudoinfarction patterns) were common in both groups, pacemaker implantation occurred only in grade 3 patients (22.2%), although this difference did not reach statistical significance. Functionally, grade 3 patients had significantly worse New York Heart Association (NYHA) functional class (P = 0.028) and shorter 6-min walk test (6MWT) distance (366 ± 38 m vs. 298 ± 110 m, *P* = 0.039). This was accompanied by significantly higher cardiac troponin I levels (0.025 ± 0.009 μg/L vs. 0.099 ± 0.09 μg/L, P < 0.001). Differences in brain natriuretic peptide (BNP) did not reach statistical significance (139 ± 82 ng/L vs. 332 ± 341 ng/L, *P* = 0.120).

Grade 3 uptake was associated with more abnormal echocardiographic parameters, including higher MWT (14.6 ± 2.3 mm vs. 16.9 ± 2.2 mm, P = 0.009), lower LVEF (53.8% ± 2.3% vs. 49.1% ± 8.2%, P = 0.003), and worse GLS (16.0% ± 4.6% vs. 12.5% ± 3.9%, P = 0.037), as well as higher rates of apical sparing (50.0% vs. 88.9%, P = 0.021). Indexed left atrial volume was similar between the groups.

### CMR data

Due to limited availability, contraindications and poor image quality in some scans, CMR was only available in 38 patients (group 2, n = 8; group 3, n = 30). CMR parameters are summarized in Table 4. The grade 3 group demonstrated significantly higher LVM index (LVMi) compared to the grade 2 group (68.1 ± 16.7 g/m^2^ vs. 106.0 ± 22.2 g/m^2^, P < 0.001). Despite this significant difference in mass, all other volumetric parameters were similar between the groups. Native T1 values tended to be higher in the grade 3 group, although this difference did not reach statistical significance (1,348.0 ± 45.9 ms vs. 1,386.0 ± 53.1 ms, P = 0.069). Furthermore, ECV was significantly higher in the grade 3 cohort compared to the grade 2 cohort (35.1% ± 4.4% vs. 45.2% ± 7.8%, P < 0.001). Among those with CMR data, grade 3 patients had higher RVFWT (4.0 ± 1.4 mm vs. 6.2 ± 1.6 mm, P = 0.001) and more frequent RV involvement, as determined by the presence of LGE (37.5% vs. 93.3%, P = 0.002).

**Table 4:** Comparison of cardiac magnetic resonance parameters between grade 2 and grade 3 groups (n = 38)

Feature	Grade 2 (n = 8)	Grade 3 (n = 30)	P-value
LVEDVi (mL/m^2^)	79.4 ± 17.2	88.2 ± 18.3	0.260
LVSVi (mL/m^2^)	42.8 ± 8.7	46.3 ± 9.7	0.390
LVEF (%)	54.7 ± 9.9	53.0 ± 7.9	0.643
RVEDVi (mL/m^2^)	69.2 ± 19.9	78.0 ± 24.5	0.386
RVSVi (mL/m^2^)	41.6 ± 9.1	43.9 ± 10.7	0.606
RVEF (%)	61.6 ± 10.0	58.2 ± 11.2	0.462
RVFWT (mm)	4.0 ± 1.4	6.2 ± 1.6	0.001^*^
RVLGE	3 (37.5)	28 (93.3)	0.002^a,*^
LAAi (cm^2^/m^2^)	16.2 ± 3.5	16.5 ± 2.3	0.810
LVMi (g/m^2^)	68.1 ± 16.7	106.0 ± 22.2	< 0.001^*^
Extracellular volume (%)	35.1 ± 4.1	45.2 ± 7.8	< 0.001^*^
Myocardial T1 (msec)	1,348.0 ± 45.9	1,386.0 ± 53.1	0.069

Values are presented as mean ± standard deviation or number (%)

LAAi, left atrial area index; LVEDVi, left ventricular end-diastolic volume index; LVEF, left ventricular ejection fraction; LVMi, left ventricular mass index; LVSVi, left ventricular stroke volume index; RVEDVi, right ventricular end-diastolic volume index; RVEF, right ventricular ejection fraction; RVFWT, right ventricular free wall thickness; RVLGE, right ventricular late gadolinium enhancement; RVSVi, right ventricular stroke volume index

^a^Calculated using the two-sided Fisher exact test

^*^P < 0.05

### Correlation analysis

Correlation analyses were performed to assess relationships among Perugini grade, clinical variables, and imaging parameters. Correlation parameters are presented in Table 5. Perugini grade demonstrated strong, significant rank-based correlations with LVMi (ρ = 0.608, *P* < 0.01) and ECV (ρ = 0.530, P < 0.01). It also correlated significantly with cardiac biomarkers, including troponin I (ρ = 0.549, *P* < 0.01) and BNP (ρ = 0.355, *P* < 0.05).

**Table 5:** Correlation matrix of key clinical, imaging, and biomarker parameters (*n* = 53)

Variable	Troponin I	BNP	6MWT	GLS^a,b^	MWT^a^	LVMi^c^	ECV
Perugini grade	0.549^**^	0.355^*^	–0.307^*^	–0.270	0.315^*^	0.608^**^	0.530^**^
Troponin I		0.140	–0.084	–0.332^*^	0.081	0.321	0.414^*^
BNP			–0.242	–0.492^**^	0.518^**^	0.478^**^	0.357^*^
6MWT				0.301^*^	–0.108	–0.144	–0.066
GLS^a,b^					–0.597^**^	–0.547^**^	–0.339^*^
MWT^a^						0.692^**^	–0.019
LVMi^c^							0.553^**^

Values represent Pearson correlation coefficients (r), except for correlations involving Perugini grade, which are Spearman rank correlation coefficients (ρ)

6MWT, 6-min walk test; BNP, brain natriuretic peptide; ECV, extracellular volume; GLS, global longitudinal strain; LVMi, left ventricular mass index; MWT, maximal wall thickness

^a^Echocardiographic data. ^b^Negative correlations with GLS reflect worsening myocardial function, as GLS values are reported as absolute values. ^c^Cardiac magnetic resonance data (n = 38)

^*^P < 0.05; ^**^P < 0.01

BNP showed significant correlations with structural and functional parameters, including a strong positive correlation with increased cardiac mass, as measured by both echocardiographic MWT (r = 0.518, P < 0.01) and CMR-derived LVMi (r = 0.478, *P* < 0.01). It was also significantly correlated with ECV (r = 0.357, P < 0.05). Functionally, higher BNP levels were strongly correlated with lower absolute GLS (r = –0.492, *P* < 0.01). There was, however, no significant correlation between BNP and troponin I (r = 0.140) or functional capacity (6MWT, r = –0.242).

Troponin I levels were significantly correlated with ECV (r = 0.414, *P* < 0.05). It was also significantly correlated with markers of cardiac function: GLS (r = –0.332, *P* < 0.05) and RVEF (r = –0.425, *P* < 0.05). However, there was no statistical association with echocardiography-derived MWT (r = 0.081) or CMR-derived LVMi (r = 0.321). Furthermore, troponin did not correlate with BNP (r = 0.140) or 6MWT (r = –0.084). Echocardiographic and CMR parameters of mass were highly correlated (r = 0.692, *P* < 0.01).

## Discussion

In this single-center study, we describe a 7-year experience with the diagnosis of ATTR-CM in a Lithuanian referral center. Our data demonstrate a substantial increase in diagnostic yield following adoption of the noninvasive diagnostic algorithm and highlight important clinical and imaging differences between patients with Perugini grade 2 and grade 3 myocardial uptake. While both grades fulfill current diagnostic criteria for ATTR-CM, grade 3 uptake was associated with clinical, biomarker, echocardiographic, and CMR markers consistent with a more advanced disease phenotype.

### Diagnostic yield

Approximately one-third of all 99mTc-PYP scans had reported cardiac-region uptake on planar imaging. However, nearly half of these were graded as equivocal or grade 1, and none of these patients were ultimately diagnosed with ATTR-CM. SPECT/CT imaging clarified that most grade 1 cases represented blood-pool activity rather than true myocardial tracer uptake, underscoring the limitations of planar imaging alone and reinforcing current guideline recommendations for SPECT/CT confirmation [12]. This finding is in line with previous reports suggesting that grade 1 uptake is less frequently associated with subsequent ATTR-CM diagnosis or adverse ATTR-CM–related outcomes [13, 14]. The overall yield of confirmed ATTR-CM was 17.5%, consistent with prior reports from referral populations [14, 15], supporting the clinical utility of targeted scintigraphic evaluation in patients with clinical or echocardiographic suspicion of amyloid cardiomyopathy. The increase in diagnoses over time likely reflects growing clinical awareness of ATTR-CM, wider use of scintigraphy-based diagnostic pathways, and the availability of disease-modifying therapy.

### Circulating biomarkers

Circulating biomarkers demonstrated distinct and complementary associations with imaging findings. BNP correlated strongly with markers of myocardial remodeling and functional impairment, including echocardiographic MWT, CMR-derived LVMi, and impaired longitudinal systolic function. In contrast, troponin I correlated with Perugini grade and ECV, but not with myocardial bulk parameters. Notably, BNP and troponin I did not correlate with each other, underscoring their reflection of different pathophysiological processes.

This dissociation is biologically plausible. Troponin release in ATTR-CM likely reflects ongoing myocardial injury driven by amyloid infiltration, microvascular dysfunction, direct cytotoxic effects, and increased wall stress at the cellular level [16, 17]. Natriuretic peptides, by contrast, predominantly reflect hemodynamic consequences of increased myocardial stiffness and chamber pressure overload [18]. Similar findings were reported by Morioka et al. [19], who observed higher troponin levels in patients with greater histological amyloid burden despite comparable natriuretic peptide levels and myocardial mass. Collectively, these findings support a multimarker approach to staging: troponin I appears to reflect the extent of infiltration and active toxicity, whereas BNP reflects the hemodynamic consequence of that infiltration.

### Associations between Perugini grade and multimodality phenotype

In our cohort, Perugini grade 3 uptake was associated with a more advanced clinical and imaging profile. Grade 3 patients had worse NYHA functional class, lower exercise tolerance, worse troponin I levels and a higher prevalence of conduction system disease, specifically wider QRS intervals and a greater need for pacemaker implantation. Structural imaging mirrored this clinical decline: echocardiography showed increased MWT and lower LVEF, while CMR showed increased LVMi, RVFWT, and expanded ECV. Additionally, both CMR and echocardiography confirmed frequent RV involvement in grade 3 patients.

Direct studies comparing detailed multimodality phenotypes between Perugini grade 2 and grade 3 ATTR-CM patients remain limited. Prior work has more commonly evaluated either the prognostic value of visual Perugini grading or quantitative/semiquantitative measures of tracer uptake. Hutt et al. [20] reported that visual Perugini grade stratification did not provide clear prognostic separation in ATTR amyloidosis, highlighting the limitations of visual grading alone. Porcari et al. [21] further showed that RV tracer uptake patterns may provide additional clinical and prognostic information beyond conventional visual grading. In contrast, quantitative scintigraphic studies have shown associations between tracer uptake and markers of disease phenotype, including echocardiographic parameters, CMR-derived ECV, biomarkers, exercise capacity, and disease stage [9, 22, 23]. Therefore, the available literature supports a relationship between scintigraphic uptake patterns and disease phenotype, but does not establish visual Perugini grade as a direct quantitative measure of myocardial amyloid burden. In this context, the present study adds a real-world PYP-based comparison of grade 2 and grade 3 patients using integrated clinical, ECG, echocardiographic, CMR, biomarker, and functional data.

### Prognostic interpretation and limitations of visual grading

Although grade 3 uptake was associated with a more advanced clinical and imaging phenotype in our cohort, these findings should not be interpreted as evidence of prognostic separation between Perugini grades. Prior studies evaluating outcomes according to visual Perugini grade have reported inconsistent results: Hutt et al. [20] found no clear prognostic separation according to visual grade, whereas Suomalainen et al. [8] suggested potential prognostic differences in patients with incidental suspected ATTR-CM on bone scintigraphy. This discrepancy may reflect the limitations of visual grading, which can be influenced by extracardiac tracer distribution, background bone uptake, technical factors, and potential ceiling effects in advanced disease [9, 20]. In contrast, quantitative and semiquantitative scintigraphic approaches may capture tracer distribution more precisely and have shown associations with disease stage and outcomes in some cohorts [9, 22]. Because outcome data were not analyzed in the present study, our findings should be interpreted as descriptive phenotype associations rather than evidence that visual Perugini grade independently predicts prognosis.

## Limitations

This study has several limitations. Its retrospective, single-center design and relatively small number of grade 2 patients limit statistical power and generalizability, although this distribution likely reflects real-world referral patterns. Given the exploratory nature of the analysis and the number of comparisons performed, no correction for multiple testing was applied; therefore, P-values should be interpreted as hypothesis-generating. Perugini grading was performed by a single experienced reader, and interobserver or intraobserver reproducibility was not assessed. Given the visual and semiquantitative nature of the Perugini scale, observer-dependent variability cannot be excluded. CMR data were not available for all patients, potentially introducing selection bias related to availability, contraindications, or image quality. BNP values were available, but N-terminal pro-BNP was not, precluding application of established staging systems. Finally, outcome data were not analyzed, preventing direct assessment of the prognostic implications of Perugini grade differences. These findings should therefore not be interpreted as demonstrating prognostic differences between Perugini grades.

## Conclusions

While Perugini grade 2 and grade 3 myocardial uptake on PYP scan both satisfy the noninvasive diagnostic criteria for ATTR-CM, they are associated with distinct clinical and imaging phenotypes. Our cohort shows that higher PYP uptake was associated with imaging markers consistent with greater myocardial involvement, more advanced functional impairment, and increased biomarkers of cardiac injury. These findings suggest that visual Perugini grade may provide descriptive phenotypic information beyond binary diagnosis in selected referral cohorts, but should not be interpreted as a direct quantitative measure of myocardial amyloid burden or as evidence of prognostic separation.

## Data Availability

The datasets used and/or analysed during the current study are available from the corresponding author on reasonable request.

## Abbreviations

6MWT: 6-Minute walk test
99mTc-PYP: Technetium-99 m pyrophosphate
ATTR-CM: Transthyretin amyloid cardiomyopathy
BNP: Brain natriuretic peptide
CMR: Cardiac magnetic resonance
CT: Computed tomography
ECG: Electrocardiogram
ECV: Extracellular volume
GLS: Global longitudinal strain
LGE: Late gadolinium enhancement
LV: Left ventricular
LVEF: Left ventricular ejection fraction
LVM: Left ventricular mass
LVMi: Left ventricular mass index
MOLLI: Modified Look-Locker inversion recovery
MWT: Maximal wall thickness
NYHA: New York Heart Association
PYP: Pyrophosphate
RV: Right ventricular
RVEF: Right ventricular ejection fraction
RVFWT: Right ventricular free wall thickness
SPECT: Single-photon emission computed tomography
